# The Effect of Different Irrigation Protocols for Smear Layer Removal on Bond Strength of a New Bioceramic Sealer

**Published:** 2013-01-20

**Authors:** Noushin Shokouhinejad, Atefeh Hoseini, Hedayat Gorjestani, Ahmad Reza Shamshiri

**Affiliations:** 1 Dental Research Center, Department of Endodontics, School of Dentistry, Tehran University of Medical Sciences, Tehran, Iran; 2 Iranian Center for Endodontic Research, Research Institute of Dental Sciences, Shahid Beheshti University of Medical Sciences, Tehran, Iran; 3 Department of Endodontics, Dental School, Shiraz University of Medical Sciences, Shiraz, Iran; 4 Oral and Dental Diseases Research Center, Dental School, Kerman University of Medical Sciences, Kerman, Iran; 5 Dental Research Center, Dental School, Tehran University of Medical Sciences, Tehran, Iran

**Keywords:** Bioceramic, Bond Strength, Root Canal Sealer, Smear Layer

## Abstract

**Introduction:**

The purpose of this study was to assess the effect of different irrigation protocols for smear layer removal on the bond strength of EndoSequence BC Sealer, a new bioceramic sealer, to root canal dentin.

**Materials and Methods:**

The middle third of forty-four extracted human teeth were sectioned horizontally to obtain 128 dentin disks. After standardization of canal spaces, dentin disks were immersed in 5.25% NaOCl for 20 min. The specimens were then randomly assigned to four groups (n=32) according to dentin treatment procedure: group 1, 17% EDTA (1 min); group 2, 17% EDTA (1 min) + 5.25% NaOCl (5 min); group 3, 17% EDTA (1 min) + 2% chlorhexidine (CHX) (5 min); and group 4, 17% EDTA (1 min) + saline (5 min). After dentin treatment, two specimens of each group were prepared for investigation with scanning electron microscopy (SEM). Surface of root canal wall was assessed in each specimen. Then the canal spaces were filled with EndoSequence BC Sealer in the remaining specimens. Push-out bond-strength and failure modes were assessed. The data on push-out test were analyzed using one-way ANOVA test. The significance level was set at P=0.05.

**Results:**

There was no significant difference between the bond strengths of test groups (P=0.203). The bond failure was mainly cohesive for all groups.

**Conclusion:**

Under the conditions of this ex vivo study, it could be concluded that the application of 17% EDTA alone or followed by 5.25% NaOCl, 2% CHX, or saline resulted in similar bond strength of EndoSequence BC Sealer to dentinal walls.

## 1. Introduction

Different types of root canal sealers based on zinc oxide, calcium hydroxide, glass ionomer, epoxy resin, silicone, and methacrylate have been introduced to endodontics [1-2]. New root canal sealers are constantly being developed for obturation of endodontically treated teeth. Recently, EndoSequence BC Sealer (Brasseler, Savannah, USA); also known as iRoot SP (Innovative Bioceramix, Vancouver, BC, Canada), has been introduced to the market. It is a bioceramic sealer based on calcium phosphate silicate [3]. It is a premixed, injectable, and hydrophilic product composed of tricalcium silicate, dicalcium silicate, calcium phosphate monobasic, calcium hydroxide, zirconium oxide which includes a similar composition to white mineral trioxide aggregate (MTA) [4]. According to the manufacturer, EndoSequence BC Sealer uses the moisture present within the dentinal tubules to initiate and complete the setting reaction. The good sealing ability of iRoot SP [4] and it’s low toxicity [5] has been shown. In addition, the bond strength of iRoot SP to radicular dentin has been reported to be equivalent to AH Plus and higher than those of Sealapex and EndoREZ [6].

Instrumentation of the root canals leaves a smear layer on the dentinal walls [7]. Root canal irrigants are used during shaping and cleaning procedures to disinfect the canal space and remove smear layer [8-9]. The question of maintaining or keeping the smear layer remains controversial [10-11]. However, the smear layer may protect the bacteria within the dentinal tubules [12] and hinder the penetration of root canal sealers into dentinal tubules [13]. It has been suggested that the mechanical interlocking of the sealer plug inside the dentinal tubules following smear layer removal may improve dislocation resistance of root filling materials [13]. A number of chemical irrigants has been evaluated to remove the smear layer. Although final irrigation of root canals with 17% ethylenediaminetetraacetic acid (EDTA) followed by 5.25% sodium hypochlorite (NaOCl) has been shown to be an effective protocol for removing the smear layer [14-16], other irrigants has been used following the application of EDTA [17-19]. Furthermore, in some studies, EDTA has been used as a final irrigant to remove the smear layer [20-21].

Chemical irrigants can alter the dentin surface composition and, therefore, affect its interaction with root canal filling materials [22]. Several studies have investigated the effect of endodontic irrigants on the bond strength of different types of root canal sealers [17, 23-25]. The high bond strength of a root canal sealer from intraradicular dentin through micromechanical retention or frictional resistance is advantageous in maintaining the integrity of the sealer-dentin interface [26-27].

It has been shown that the bond strength value of gutta-percha combined with EndoSequence BC Sealer after removing the smear layer using EDTA followed by NaOCl was not different from the bond strength of filling material in the presence of smear layer [28]. However, to the best of our knowledge, no information is available on the effect of different protocols for smear layer removal on bond strength of new calcium phosphate silicate-based sealers to intraradicular dentin. This study used a push-out test to assess the bond strength of EndoSequence BC Sealer to root canal dentin after smear layer removal with different protocols.

## 2. Material and Methods

Forty-four extracted human teeth were sectioned below the cement-enamel junction. The roots were embedded in acrylic resin and then were sectioned horizontally to provide one-hundred and twenty eight 2-mm thick dentin disks from the middle third of the roots. Standardized simulated canal spaces were prepared with a tapered bur (larger diameter = 2.70 mm; smaller diameter = 2.30 mm; length = 2 mm). Dentin disks were immersed in 5.25% NaOCl for 20 min to simulate the irrigation during root canal preparation and then, according to the dentin treatment, were randomly divided into 4 groups (n = 32) as follows:

Group 1. (EDTA): Thirty two dentin disks were immersed in 17% EDTA (Vista Dental, Racine, US) for 1 min.

Group 2. (EDTA/NaOCl): Specimens were first immersed in 17% EDTA for 1 min and then in 5.25% NaOCl for 5 min.

Group 3. (EDTA/CHX): Dentin disks were immersed in 17% EDTA for 1 min followed by 2% CHX (Consepsis, Ultradent, South, Jordan, UT) for 5 min.

Group 4 (EDTA/Saline): Dentin disks were immersed in 17% EDTA for 1 min and then finally immersed in saline for 5 min.

After dentin treatment, two specimens of each group were prepared for scanning electron microscopy (SEM) to examine the root canal wall after the use of each irrigation protocol. Dentin disks were split longitudinally along the center of the canal. The pulpal walls of the specimens were mounted on aluminium stubs, sputter coated with gold, and examined under a SEM (Vega II XMU, Tescan, Czech Republic) at 15 kV to evaluate the root canal wall.

The other 30 specimens in each group were used for push-out test. Standardized canal spaces were dried with paper points and filled with EndoSequence BC Sealer (Brasseler, Savannah, USA). Specimens were then stored at 37ºC and 95% humidity for 7 days to allow the sealer to set.

Push-out Test

The filling material was loaded with a 2 mm diameter cylindrical stainless-steel plunger. Loading was performed on a universal testing machine (Z050, Zwick/Roell, Ulm, Germany) at a speed of 0.5 mm/min until debonding occurred. The load was applied in an apical-coronal direction to avoid any interference because of the root canal taper. The bond strength value in megapascals (MPa) was computed by dividing the maximum load needed to dislodge the filling material in Newtons by the interfacial area (mm^2^). Sealer-dentin interfacial area was calculated by 0.5× (circumference of coronal aspect of standardized canal space + circumference of apical aspect) × thickness of the dentin disk [29].

Failure Mode Analysis

After the push-out test, the specimens were examined under optical magnification (×25) to determine modes of failure: adhesive at the filling material-dentin interface, cohesive within filling material, and mixed failure.

The data on push-out test were analyzed using one-way ANOVA test. The significance level was set at *P*=0.05.

## 3. Results

The mean ± standard deviation values (in MPa) of push-out bond strength of EndoSequence BC Sealer for each group are shown in Table 1. No significant difference were found between the groups (*P*=0.203). Failure analysis showed the predominant failure modes to be cohesive for all groups.

**Table 1.** Push-out bond strength values [mean (SD)] in MPa for the experimental groups

Group (n=30)	Bond strength			
	Mean	Min	Max	Range
**Group 1: EDTA**	1.8 (1.05)	0.17	4.23	4.06
**Group 2: EDTA +NaOCl**	1.5 (0.81)	0.37	3.18	2.81
**Group 3: EDTA + CHX**	1.6 (1.1)	0.26	3.75	3.49
**Group 4: EDTA + Saline**	1.5 (0.79)	0.38	3.36	2.98

**Figure 1.**
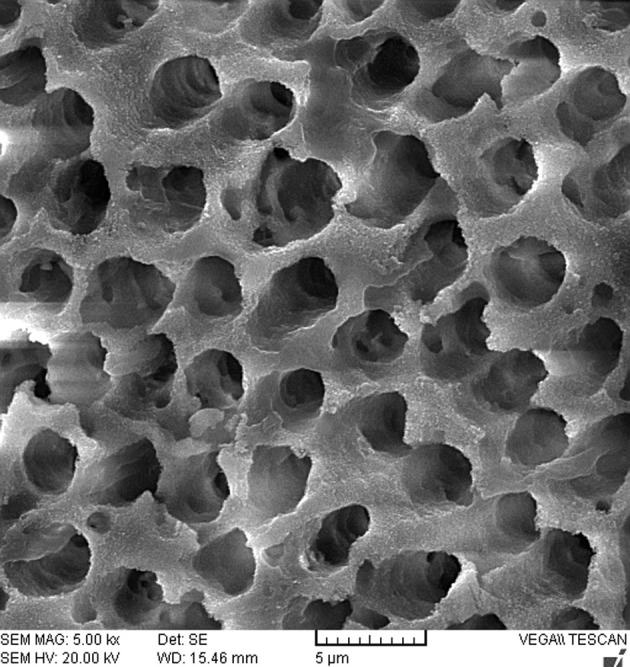
SEM analysis of dentinal walls with no smear layer and open tubule orifices

The SEM analysis of dentinal walls after treatment of dentin disks in all groups revealed open tubule orifices with the absence of smear layer (Figure 1).

## 4. Discussion

The results of this study showed that there was no significant difference in dislocation resistance of EndoSequence BC Sealer after different dentin treatments. A variety of irrigants has been used for removing the smear layer after EDTA use [15-19]. In this study, in order to simulation the irrigation performed during canal preparation and for maximum removal of the organic component of the smear layer, the dentin disks were initially immersed in 5.25% NaOCl for 20min. In the present study, SEM examination of pulpal walls revealed that the use of 17% EDTA for 1 min alone or followed by 5.25% NaOCl, 2% CHX, or saline was effective in complete removal of the smear layer. This is in agreement with Moon *et al.* [20] and Parirokh *et al*. [30] who showed the effectiveness of a 1-min irrigation of 17% EDTA on smear layer removal. In addition, the finding of this study is consistent with Menezes *et al.* who showed the use of 2% chlorhexidine solution combined to 17% EDTA promoted an effective cleaning of dentinal walls [31]. It has been stated that EndoSequence BC Sealer has the same composition to white MTA [4]. Although no information is available with regard to the influence of chemical irrigants on EndoSequence BC Sealer, several studies have examined the effects of endodontic irrigants on physicochemical properties of MTA [32-34]. Lee *et al.* showed the adverse effect of EDTA on hydration and microhardness of MTA and stated that the residual EDTA in the root canal system may chelate calcium ions released from MTA during hydration, thereby interfere with the precipitation of hydrated products [34]. However, Nandini *et al.* showed that 17% EDTA had no effect on surface hardness of 1 and 21-day specimens of white ProRoot MTA, but significant surface dissolution of 1-day set MTA was observed after exposure to 2% CHX [35]. Another study regarding the effect of chemical irrigants on bond strength of MTA from dentin disks showed that the immersion in 5.25% NaOCl and 2% CHX for 2 hours had no adverse effect on dentin-bond strength of MTA [32].

In the present study, EndoSequence BC Sealer was contacted with only dentinal walls treated with different endodontic irrigants and dried before filling. Therefore, the comparison of the results of this study to those of aforementioned studies [32-34] which the surface of MTA was completely exposed to chemical irrigants is difficult.

Based on the results of this study, final irrigation of the dentin disks with 2% CHX and 5.25% NaOCl did not affect the bond strength of EndoSequence BC Sealer. Although chlorhexidine and NaOCl lack the ability to remove the smear layer [31, 36], these antimicrobial solutions have been suggested to be used as the final rinses for canal disinfection [9, 37-38].

Analysis of failure mode showed the predominant failure modes to be cohesive failure. This finding is in agreement with Ersahan and Aydin who revealed the mode of failure was mainly cohesive for iRoot SP sealer [6]. Furthermore, a previous study showed the bond failure to be predominantly cohesive for EndoSequence BC Sealer combined with gutta-percha [28].

In dynamic clinical situations, adhesion is necessary to avoid dislocation of sealer because of tooth flexure, operative procedures, or post space preparation [26, 39]. However, it is not certain that greater filling material adhesion will result in higher clinical success [39-40].

## 5. Conclusion

Under the conditions of this *ex vivo* study, it could be concluded that using 17% EDTA alone or followed by 5.25% NaOCl, 2% CHX, or saline for removing the smear layer resulted in similar bond strength for EndoSequence BC Sealer. It is important to mention that further investigations should be conducted to evaluate the effect of different irrigants on clinical success of roots filled with EndoSequence BC Sealer.
